# α-Synuclein Degradation in Brain Pericytes Is Mediated via Akt, ERK, and p38 MAPK Signaling Pathways

**DOI:** 10.3390/ijms26041615

**Published:** 2025-02-14

**Authors:** Miki Yokoya, Fuyuko Takata, Takuro Iwao, Junichi Matsumoto, Yasuyoshi Tanaka, Hisataka Aridome, Miho Yasunaga, Junko Mizoguchi, Kazunori Sano, Shinya Dohgu

**Affiliations:** 1 Department of Pharmaceutical Care and Health Sciences, Faculty of Pharmaceutical Sciences, Fukuoka University, Fukuoka 814-0180, Japan; pd211011@cis.fukuoka-u.ac.jp (M.Y.); ftakata@fukuoka-u.ac.jp (F.T.); t.iwao.ot@adm.fukuoka-u.ac.jp (T.I.); jmatsumoto@fukuoka-u.ac.jp (J.M.); yasutanaka@fukuoka-u.ac.jp (Y.T.); pd191001@cis.fukuoka-u.ac.jp (H.A.); pd211010@cis.fukuoka-u.ac.jp (M.Y.); pd231004@cis.fukuoka-u.ac.jp (J.M.); 2 Department of Physiology and Pharmacology, Faculty of Pharmaceutical Sciences, Fukuoka University, Fukuoka 814-0180, Japan; ksano@fukuoka-u.ac.jp

**Keywords:** alpha-Synuclein, pericyte, Parkinson’s disease, blood–brain barrier, autophagy, ubiquitin–proteasome system, Akt, ERK, p38 MAPK

## Abstract

Parkinson’s disease (PD) is characterized by widespread distribution of Lewy bodies, which are composed of phosphorylated and aggregated forms of α-Synuclein (α-Syn), in the brain. Although the accumulation and propagation of α-Syn contribute to the development of PD, the involvement of the blood–brain barrier (BBB) in these processes remains unknown. Pericytes, one of the cell types that constitute the BBB, degrade various forms of α-Syn. However, the detailed mechanisms involved in α-Syn degradation by pericytes remain poorly understood. Therefore, in this study, we aimed to determine the ability of the BBB-constituting cells, particularly primary cultures of rat pericytes, brain endothelial cells, and astrocytes, to degrade α-Syn. After α-Syn uptake by the cells, intracellular α-Syn decreased only in pericytes. This pericyte-specific α-Syn decrease was inhibited by an autophagy inhibitor, bafilomycin A1, and a proteasome inhibitor, MG132. siRNA-mediated knockdown of degradation enzymes or familial PD-associated genes, including cathepsin D, DJ-1, and LRRK2, did not affect α-Syn clearance in pericytes. However, pharmacological inhibitors of Akt, ERK, and p38 MAPK inhibited α-Syn degradation by pericytes. In conclusion, our results suggest that α-Syn degradation by pericytes is mediated by an autophagy–lysosome system and a ubiquitin–proteasome system via α-Syn-activated Akt, ERK, and p38 MAPK signaling pathways.

## 1. Introduction

Parkinson’s disease (PD) is a progressive neurodegenerative disease characterized by the aggregation and accumulation of α-Synuclein (α-Syn), a protein involved in synaptic function and neuronal survival, in the brain [1]. The neuropathological hallmark of PD includes the progressive loss of substantia nigra dopaminergic neurons and presence of Lewy bodies [2,3]. The loss of dopaminergic neurons leads to dopamine depletion, which is a major cause of PD motor symptoms (bradykinesia, tremor, rigidity, and postural instability). The primary therapy for PD is dopamine replacement therapy, with levodopa serving as the gold standard for PD treatment. However, owing to the incomplete understanding of PD pathogenesis, there is currently no treatment that can stop or hinder PD progression.

Aggregation of α-Syn, a small protein (14 kDa) that predominantly exists as a soluble monomer [2,4], is associated with PD. Genetic and environmental factors modulate monomeric α-Syn aggregation [5,6]. These factors contribute to the formation of insoluble and highly toxic α-Syn aggregates, such as oligomers, fibrils, and ribbons, leading to the formation of Lewy bodies [7,8]. The progressive accumulation of α-Syn aggregates leads to neurodegeneration and neuronal cell death [9,10]. In addition to α-Syn aggregation, several postmortem studies have shown that tau is co-localized with α-Syn in Lewy bodies in PD brains, suggesting that tauopathies are associated with the pathogenesis of PD [11]. Neuroinflammation contributes to the pathophysiology of PD and other Parkinsonian syndromes, including multiple system atrophy (MSA) and progressive supranuclear palsy (PSP) [12,13,14]. Differences in inflammatory factors have been revealed in various Parkinsonian syndromes [13,15]. Microglial activation has been observed in the brains of patients with these synucleinopathies and tauopathies. Neurodegeneration and cell death in these diseases are partly caused by inflammatory responses due to exposure to pathogenic proteins, such as aggregated α-Syn and tau. These pathogenic proteins also induce microglial activation to release various inflammatory cytokines [12,16]. In turn, activated microglia facilitate α-Syn aggregation, oligodendrocyte apoptosis in MSA, and tau deposition in PSP [12,13,14]. In addition, a recent study revealed that α-Syn-mediated neurotoxicity is induced by the interaction of tissue plasminogen activator with N-methyl-D-aspartate receptor 1 [17]. Thus, the removal of α-Syn aggregates and the prevention of their formation are considered promising disease-modifying strategies for synucleinopathies, such as PD.

α-Syn is a neuronal protein that predominantly localizes to presynaptic nerve terminals [18,19]. α-Syn is propagated from neuron to neuron through tunneling nanotubes or extracellular release [20,21,22] as well as between neuron and glial cells, such as astrocytes and microglia [23,24,25]. Normally, these cells degrade intracellular α-Syn via the autophagy–lysosome pathway (ALP) and the ubiquitin–proteasome system (UPS) [26,27,28,29,30,31]. However, lysosome and proteasome activity tend to decline with age, leading to α-Syn accumulation in the brain [32,33,34,35]. Dysfunction of these degradation systems and α-Syn inclusions in neurons and glial cells have been observed in the brain of patients with PD [36,37,38], suggesting that disruption of the α-Syn clearance system in the brain is involved in the pathogenesis of PD.

In addition to neurons and glial cells, pericytes, which are located at the wall of cerebral capillaries, take up and degrade α-Syn aggregates [39,40]. In our previous in vitro study, we showed that pericytes take up α-Syn and then release various inflammatory mediators to induce blood–brain barrier (BBB) dysfunction [41]. The location of pericytes enables these cells to receive α-Syn through two primary pathways: (1) direct transfer from neurons and glial cells through tunneling nanotubes and extracellular release [42,43] and (2) transport from circulating blood across the BBB [44]. BBB disruption may contribute to blood-borne α-Syn penetration into the brain [45]. Furthermore, in PD brains, α-Syn aggregates are present in pericytes, microglia, and astrocytes [46], indicating that α-Syn degradation in pericytes is impaired in PD.

Although it is clear that pericytes possess α-Syn phagocytic or degradative abilities, the detailed molecular mechanisms remain unclear. Therefore, in this study, we aimed to determine the pericyte-driven mechanism of α-Syn clearance and elucidate the involvement of pericytes in PD pathogenesis. Furthermore, we aimed to identify the molecules involved in α-Syn clearance by pericytes.

## 2. Results

### 2.1. Time-Course of Oligomeric and Monomeric α-Syn Uptake by Pericytes, Brain Endothelial Cells, and Astrocytes

**Figure 1:**
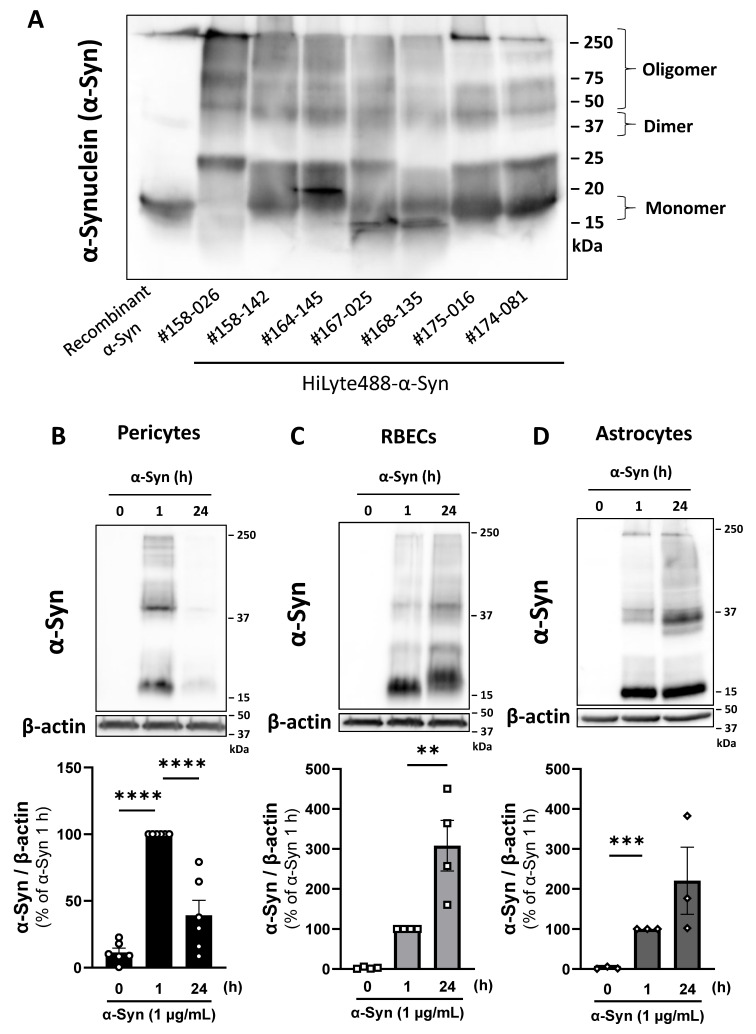
Time-course of α-Syn uptake by pericytes, RBECs, and astrocytes. (**A**) Representative Western blot images showing the molecular weight of α-Syn included in recombinant human α-Syn and each lot of HiLyte488-α-Syn (HL-α-Syn; AnaSpec Inc.). α-Syn (1 μg/lane) was loaded on SDS-PAGE, and α-Syn immunoreactivities were detected with an anti-α-synuclein antibody (MJFR1). (**B**–**D**) Intracellular α-Syn in pericytes (**B**), RBECs (**C**), and astrocytes (**D**) at 0, 1, and 24 h after HL-α-Syn (1 μg/mL) treatment. The top of each panel shows representative Western blots of α-Syn (~17 kDa) in cell lysates. Cell lysates were loaded on SDS-PAGE, and α-Syn was detected with an anti-α-synuclein antibody (MJFR1). β-actin was used as a loading control. The lower panel shows quantitative analysis of Western blots using densitometry. The results are expressed as the percentage of each corresponding α-Syn level at 1 h. Values are the means ± SEM (n = 3–6). Statistical analysis was performed using Dunnett’s multiple comparisons test. (** *p* < 0.01, *** *p* < 0.001, **** *p* < 0.0001).

Western blot analysis (Figure 1A) showed that recombinant human α-Syn had a 17 kDa band, indicating that recombinant α-Syn contained mainly monomeric α-Syn (17 kDa). Several lots of HiLyte488-conjugated human recombinant α-Synuclein (HL-α-Syn, AnaSpec Inc., Fremont, CA, USA) exhibited a broad band, containing both monomeric (17 kDa) and oligomeric α-Syn (>17 kDa; Figure 1A). Therefore, in this study, we employed HL-α-Syn to evaluate the monomeric and oligomeric α-Syn uptake ability of BBB-constituting cell types (pericytes, rat brain endothelial cells (RBECs), and astrocytes) using Western blot.

The uptake of α-Syn by pericytes, RBECs, and astrocytes was evaluated over time. The cells were HL-α-Syn (1 μg/mL) treated for 24 h. Pericytes took up oligomeric and monomeric α-Syn within 1 h. Following HL-α-Syn exposure, intracellular levels of oligomeric and monomeric α-Syn in pericytes at 24 h were significantly lower (60%) than those at 1 h (Figure 1B). Intracellular α-Syn levels increased in RBECs and astrocytes with increasing time. Following HL-α-Syn exposure, α-Syn levels in RBECs and astrocytes at 24 h were four- and two-fold higher than those at 1 h (Figure 1C,D).

### 2.2. Degradation of α-Syn in Pericytes Is Mediated Through the Autophagy–Lysosome Pathway and Ubiquitin–Proteasome System

**Figure 2:**
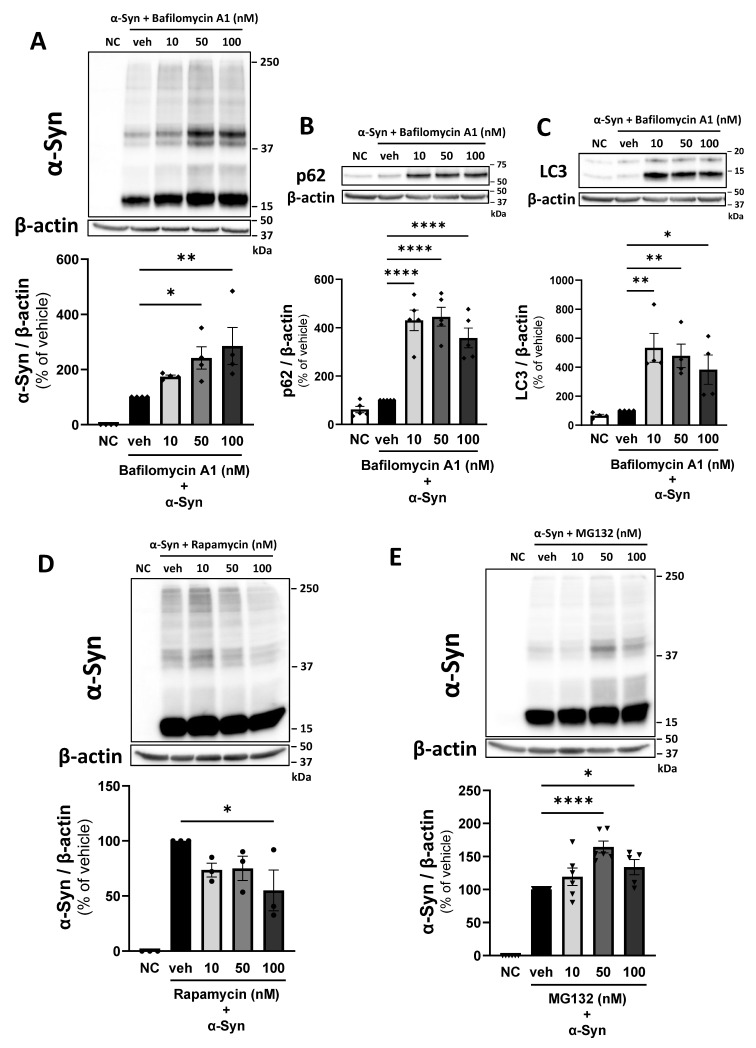
α-Syn degraded in pericytes through the autophagy–lysosome pathway and ubiquitin–proteasome system. (**A**–**C**) Effect of bafilomycin A1 on intracellular α-Syn (**A**) and expression of p62 (**B**) and LC3 (**C**) in pericytes. (**D**) Effect of rapamycin on intracellular α-Syn in pericytes. (**E**) Effect of MG132 on intracellular α-Syn in pericytes. Pericytes were treated with HL-α-Syn (1 µg/mL) in the presence of bafilomycin A1 (autophagy–lysosome inhibitor; 10–100 nM) (**A**–**C**), rapamycin (autophagy inducer; 10–100 nM) (**D**), and MG132 (ubiquitin–proteasome inhibitor; 10–100 nM) (**E**) for 24 h. Each panel shows representative Western blot images and quantitative analysis. β-actin was used as a loading control. The results are expressed as the percentage of the corresponding vehicle-treated pericytes, which were incubated with HL-α-Syn alone for 24 h. NC indicates non-treated pericytes as a negative control. Values are the means ± SEM (n = 3–6). Statistical analysis was performed using Dunnett’s multiple comparisons test. (* *p* < 0.05, ** *p* < 0.01, **** *p* < 0.0001).

To determine whether the decrease in α-Syn in pericytes at 24 h after exposure was due to α-Syn degradation via autophagy and the ubiquitin–proteasome pathway, pericytes were treated with HL-α-Syn in the presence of bafilomycin A1 (lysosome–autophagy inhibitor) or MG132 (ubiquitin–proteasome inhibitor) for 24 h. Bafilomycin A1 (50 and 100 nM) significantly increased both oligomeric and monomeric α-Syn in pericytes (241.9 and 285.3% of vehicle, respectively; Figure 2A). The autophagy markers p62 and LC3 significantly increased in pericytes treated with 10 to 100 nM bafilomycin A1 (p62; 430.7, 445.4, and 357.8%, LC3; 534.2, 478.8, and 383.6% of vehicle; Figure 2B,C). To further investigate the involvement of autophagy in α-Syn degradation by pericytes, pericytes were treated with HL-α-Syn and an autophagy promoter, rapamycin (10–100 nM), for 24 h. Rapamycin (100 nM) significantly decreased α-Syn in pericytes (55.01% of vehicle; Figure 2D). Furthermore, pericytes were treated with the ubiquitin–proteasome inhibitor MG132 (50 and 100 nM), resulting in a significant increase in monomeric and oligomeric α-Syn in pericytes during 24 h exposure to HL-α-Syn (164.3 and 133.8% of vehicle; Figure 2E). These results suggest that the degradation of monomeric and oligomeric α-Syn by pericytes is mediated by autophagy and the ubiquitin–proteasome system.

### 2.3. Pericytes Decrease Internalized α-Syn Intracellularly via Degradation

**Figure 3:**
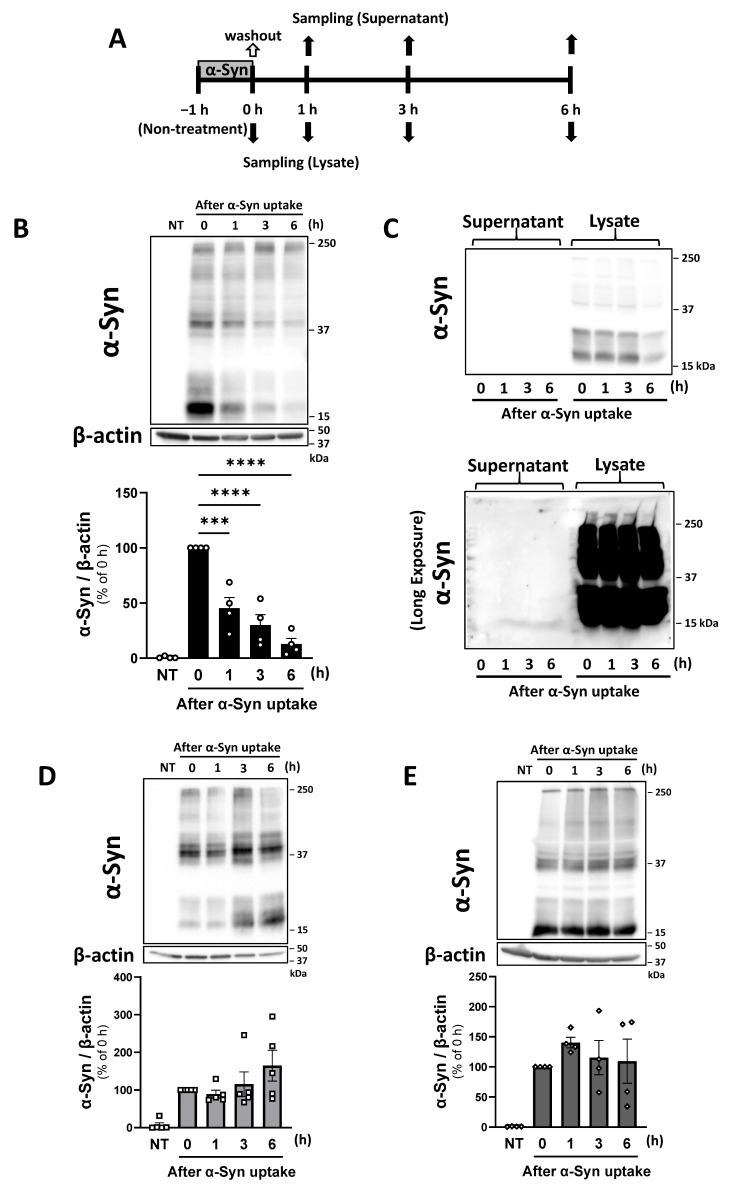
Internalized α-Syn in pericytes decreased in a time-dependent manner. (**A**) Experimental procedures for evaluating degradation and extracellular release of α-Syn. Cells were incubated with HL-α-Syn (1 µg/mL) for 1 h and subsequently washed twice with fresh physiological buffer. Cells were then incubated in fresh physiological buffer for the indicated time up to 6 h. Culture supernatants were collected to measure extracellular release. (**B**–**E**) α-Syn levels in cell lysates obtained from pericytes (**B**), RBECs (**D**), and astrocytes (**E**). Each panel shows representative Western blot images and quantitative analysis. β-actin was used as a loading control. NT indicates non-treated cells as a negative control. The results are expressed as the percentage of each corresponding control (0 h), which are cells after a 1 h HL-α-Syn uptake. Values are the means ± SEM (n = 4–5). Statistical analysis was performed using Dunnett’s multiple comparisons test (*** *p* < 0.001, **** *p* < 0.0001). (**C**) Representative Western blot of α-Syn levels in culture supernatants obtained from pericytes. Aliquots of supernatants (350 μL out of 1 mL) were concentrated to approximately 25 μL using an Amicon Ultra centrifugal filter (3 kDa cut-off). Aliquots of lysates (35 μL of 100 μL) obtained from pericytes were loaded as a positive control. The bottom in the panel shows representative Western blot images with an extended exposure to detect immunoreactive bands for α-Syn. No immunoreactive bands were detected in culture supernatants.

To assess whether pericytes secreted intracellular α-Syn into the extracellular space, we measured α-Syn levels in both cell lysates and culture supernatants up to 6 h following 1 h incubation with HL-α-Syn (Figure 3A). One hour after HL-α-Syn exposure, intracellular α-Syn in pericytes decreased significantly in a time-dependent manner (1 h: 45.31%, 3 h: 29.90%, 6 h: 12.79% of 0 h; Figure 3B). However, α-Syn was not detected in pericyte-derived supernatants, regardless of exposure time (Figure 3C). Intracellular levels of α-Syn in RBECs and astrocytes did not decrease (Figure 3D,E). These findings suggest that α-Syn uptake by pericytes and subsequent α-Syn clearing occur via the protein degradation system rather than through extracellular secretion.

**Figure 4:**
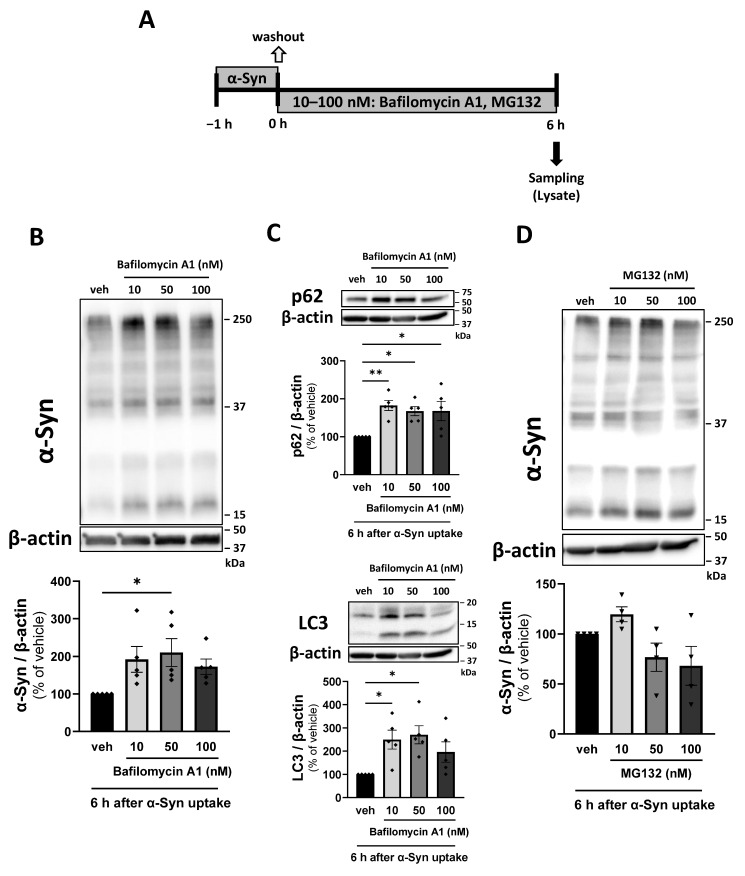
Effect of bafilomycin A1 and MG132 on the rapid decrease in internalized α-Syn in pericytes 0–6 h after α-Syn uptake. (**A**) Experimental procedures for investigating the degradation system of α-Syn in pericytes 0–6 h after α-Syn uptake. Pericytes were incubated with HL-α-Syn (1 µg/mL) for 1 h and subsequently washed twice with fresh physiological buffer. Pericytes were then incubated in fresh physiological buffer containing bafilomycin A1 (10–100 nM) (**B**,**C**) and MG132 (10–100 nM) (**D**) for 6 h. (**B**,**C**) Intracellular α-Syn (**B**), p62 (**C**), and LC3 (**C**) levels in pericytes treated with bafilomycin A1 for 6 h after α-Syn uptake. (**D**) Intracellular α-Syn levels in pericytes treated with MG132 for 6 h after α-Syn uptake. Each panel shows representative Western blot images and quantitative analysis. β-actin was used as a loading control. The results are expressed as the percentage of vehicle-treated pericytes. Values are the means ± SEM (n = 4–5). Statistical analysis was performed using Dunnett’s multiple comparisons test. (* *p* < 0.05, ** *p* < 0.01).

To confirm whether the decrease in intracellular α-Syn following α-Syn uptake over 6 h is inhibited by bafilomycin A1 and MG132, pericytes were treated with bafilomycin A1 (10–100 nM) and MG132 (10–100 nM) for 6 h, as illustrated in the schematic diagram (Figure 4A). Oligomeric and monomeric α-Syn in pericytes increased significantly in the presence of 50 nM bafilomycin A1 (210.4% of vehicle; Figure 4B). Additionally, the autophagy markers p62 and LC3 led to a significant increase in α-Syn in pericytes (p62; 167.5%, LC3; 270.4% of vehicle; Figure 4C). However, MG132 did not increase α-Syn in pericytes (Figure 4D).

### 2.4. Cathepsin D, DJ-1, and Lrrk2 Are Not Involved in α-Syn Degradation by Pericytes

**Figure 5:**
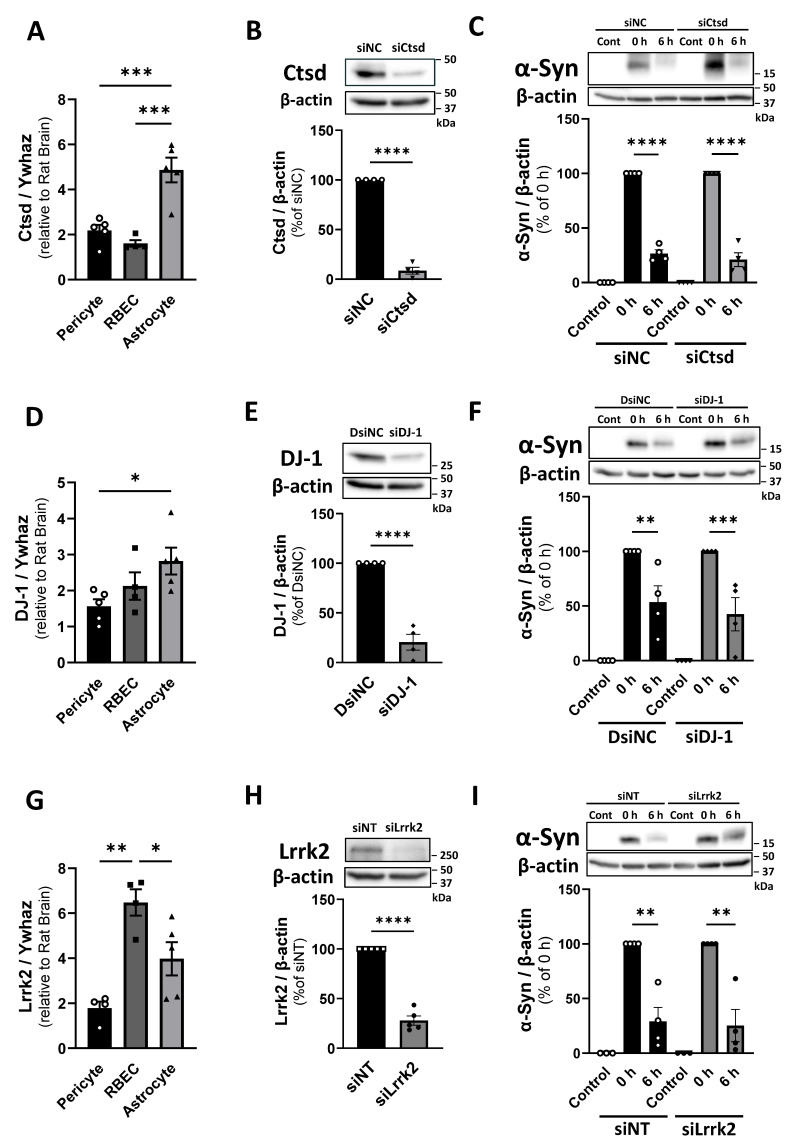
Cathepsin D, DJ-1, and Lrrk2 do not mediate α-Syn degradation in pericytes. (**A**,**D**,**G**) mRNA expression levels of Ctsd (**A**), DJ-1 (**D**), and Lrrk2 (**G**) in pericytes, RBECs, and astrocytes. mRNA levels measured by RT-qPCR using the *Ywhaz* gene as the reference gene for normalization. The results are expressed as fold changes relative to a calibrator (total RNA derived from rat brain). (**B**,**E**,**H**) siRNA-mediated knockdown of cathepsin D (**B**), DJ-1 (**E**), and Lrrk2 (**H**) in pericytes. Pericytes were transfected with negative control siRNA (siNC, DsiNC, and siNT) or siRNA targeting cathepsin D (siCtsd), DJ-1 (siDJ-1), and Lrrk2 (siLrrk2) for 72 h. The results are expressed as the percentage of negative control siRNA-transfected pericytes. (**C**,**F**,**I**) Intracellular monomeric α-Syn in pericytes transfected with negative control siRNA or siRNA targeting cathepsin D (**C**), DJ-1 (**F**), and Lrrk2 (**I**) after α-Syn uptake. After 72 h of siRNA transfection, pericytes were incubated with α-Syn (1 µg/mL) for 1 h and subsequently washed twice with fresh physiological buffer. Pericytes were then incubated in fresh physiological buffer for 6 h. β-actin was used as a loading control. Each panel shows representative Western blot images and quantitative analysis. The results are expressed as the percentage of the corresponding siRNA-transfected pericytes after 1 h α-Syn uptake (0 h). Control indicates pericytes without α-Syn incubation as a negative control. Values are the means ± SEM (n = 3–5). Statistical analysis was performed using Student’s *t*-test or one-way ANOVA, followed by the Tukey–Kramer test. (* *p* < 0.05, ** *p* < 0.01, *** *p* < 0.001, **** *p* < 0.0001).

To explore candidate molecules involved in α-Syn degradation in pericytes, we focused on cathepsin D (*Ctsd*), the major lysosomal protease involved in α-Syn degradation [47], and the familial PD-related genes DJ-1 (*Park7*) and Leucine-rich repeat kinase2 (*Lrrk2*). mRNA expression of Ctsd, DJ-1, and Lrrk2 was observed in BBB-constituting cell types (Figure 5A,D,G). Treatment of pericytes with siRNAs for Ctsd, DJ-1, and Lrrk2 significantly reduced the expression of each corresponding molecule (Figure 5B,E,H). However, Ctsd, DJ-1, and Lrrk2 knockdown did not alter α-Syn degradation activity in pericytes (Figure 5C,F,I).

### 2.5. Pericytes Degrade α-Syn Through Akt, ERK, and p38 MAPK Pathway Activation

**Figure 6:**
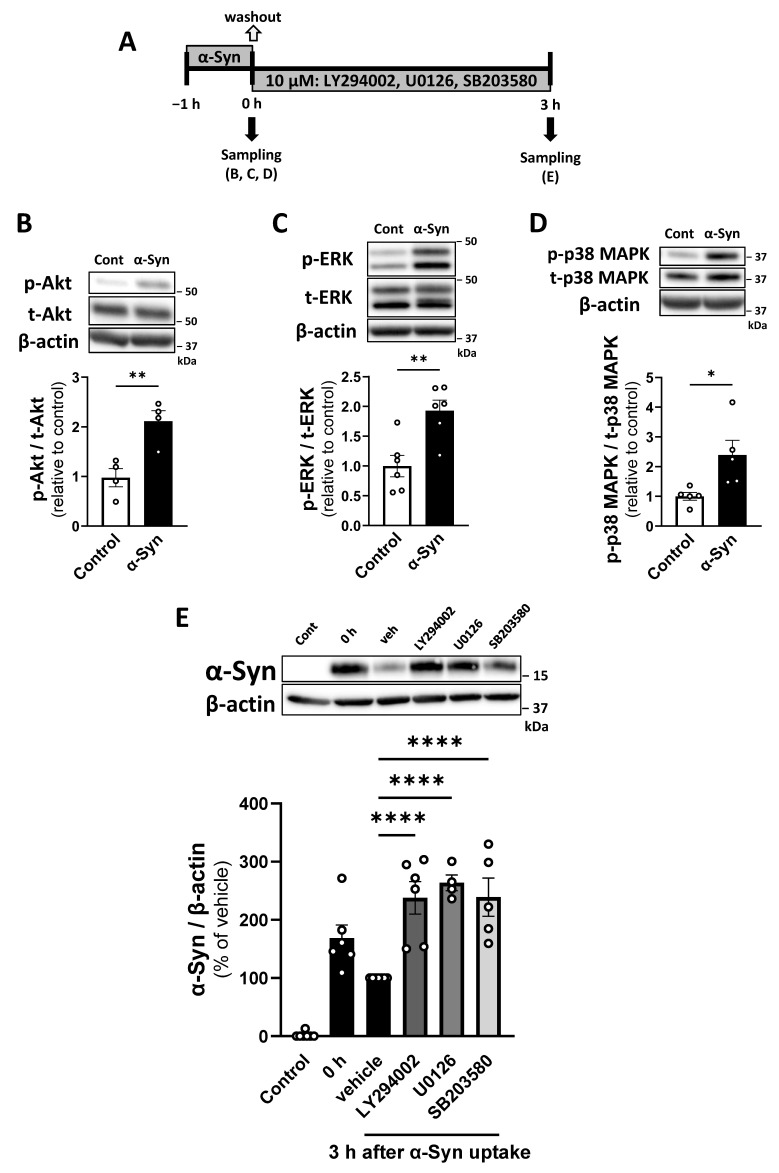
α-Syn degradation in pericytes is mediated by Akt, ERK, and p38 MAPK signaling pathways. (**A**) Experimental procedures for investigating the involvement of signaling pathways in degradation of α-Syn by pericytes 3 h after α-Syn uptake. Pericytes were incubated with recombinant human α-Syn (1 µg/mL) for 1 h and subsequently washed twice with fresh physiological buffer. Pericytes were then incubated in fresh physiological buffer containing LY294002 (10 μM), U0126 (10 μM), and SB203580 (10 μM) for 3 h. Pericytes with or without 1 h of α-Syn incubation were used as a positive (defined as “0 h”) and negative control (defined as “control”), respectively. (**B**–**D**) Phosphorylation levels of Akt (**B**), ERK (**C**), and p38 MAPK (**D**) in pericytes treated with α-Syn. Pericytes were incubated with recombinant human α-Syn (1 µg/mL) for 1 h. Each panel shows representative Western blot images of total and phosphorylated protein and quantitative analysis of the relative ratio of phosphorylated protein to total protein. β-actin was used as a loading control. (**E**) Representative Western blot images of intracellular α-Syn levels in pericytes in the absence or presence of LY294002, U0126, and SB203580 3 h after 1 h α-Syn uptake. β-actin was used as a loading control. The bar graph in the bottom shows quantitative analysis. The results are expressed as the percentage of vehicle-treated pericytes 3 h after 1 h α-Syn uptake. Values are the means ± SEM (n = 4–7). Statistical analysis was performed using Student’s *t*-test or one-way ANOVA, followed by Dunnett’s multiple comparisons test. (* *p* < 0.05, ** *p* < 0.01, **** *p* < 0.0001).

We determined which pathways were rapidly activated by α-Syn in pericytes to determine intracellular signaling pathways leading to α-Syn degradation in pericytes. Phosphorylation levels of Akt, ERK, and p38 MAPK in pericytes increased significantly 1 h after α-Syn exposure (Figure 6B–D). Next, we determined whether the inhibition of these pathways, using pharmacological inhibitors (LY294002, U0126, and SB203580), attenuated α-Syn degradation in pericytes. Pericytes were treated with these inhibitors during the degradation phase after α-Syn uptake (1 h) to exclude the possibility that these inhibitors affect α-Syn uptake by pericytes. Intracellular α-Syn levels in vehicle-treated pericytes decreased 3 h after 1 h α-Syn uptake. Intracellular α-Syn in LY294002-, U0126-, and SB203580-treated pericytes was significantly higher than that in vehicle-treated pericytes (237.8, 263.7, and 239.1% of vehicle, respectively; Figure 6E).

## 3. Discussion

To date, no disease-modifying therapies or preventive interventions targeting the elimination of the pathogenic protein α-Syn have been established. Although α-Syn interacts with the BBB and spreads between cells during the progression of PD, there is limited evidence on α-Syn uptake and degradation processes at the BBB, particularly as a potential α-Syn clearance mechanism. This study provides novel insights, demonstrating that α-Syn clearance is pericyte-specific at the BBB, by comparing α-Syn clearance across different BBB-constituting cell types. We demonstrated that pericytes degrade α-Syn without releasing it into the extracellular space. Furthermore, we identified the intracellular signaling pathways involved in the α-Syn degradation system within pericytes.

We found that commercially available HL-α-Syn contains α-Syn of various molecular weights. Therefore, we employed HL-α-Syn as monomeric and oligomeric α-Syn to evaluate the α-Syn uptake ability of BBB-constituting cell types (pericytes, brain endothelial cells, and astrocytes). Intracellular levels of oligomeric and monomeric α-Syn in all cells increased 1 h after exposure, indicating that all cells possessed the ability to take up α-Syn. Twenty-four hours after α-Syn exposure, unlike RBECs and astrocytes, pericytes exhibited decreased intracellular α-Syn levels. These findings suggest that, among the BBB-constituting cells, pericytes possess the unique ability to clear α-Syn. In contrast to previously reported findings [48], we were unable to confirm α-Syn clearance by astrocytes during 24 h. This study is the first to demonstrate that the α-Syn internalization profile varies among different BBB-constituting cells. Our findings suggest that pericytes clear α-Syn more rapidly than astrocytes and brain endothelial cells.

Our findings are consistent with recent studies showing that pericytes take up and degrade α-Syn aggregates [39]. However, there is little experimental evidence regarding the mechanisms underlying α-Syn clearance by pericytes. To elucidate these mechanisms, we first focused on the autophagy–lysosome pathway (ALP) and ubiquitin–proteasome system (UPS), which are major protein degradation pathways. This focus was based on previous findings that microglia, a key cell type in the brain [31,49,50], degrade α-Syn received from neurons via the ALP. We found that both oligomeric and monomeric α-Syn and the autophagy markers p62 and LC3 increased significantly when pericytes were treated with bafilomycin A1, an autophagy inhibitor, for 24 h. This suggests that bafilomycin A1 effectively inhibited autophagy activity in pericytes, leading to the increase in intracellular α-Syn. Moreover, these results were supported by rapamycin, an autophagy inducer, causing a significant decrease in monomeric and oligomeric α-Syn levels in pericytes. In addition, when UPS, a protein degradation pathway, was inhibited using MG132, intracellular α-Syn levels in pericytes increased after 24 h. These results suggest that the decrease in α-Syn levels in pericytes observed during 24 h α-Syn incubation was due to degradation through the ALP and UPS.

These results, showing a decrease in α-Syn in pericytes, were obtained by continuously treating cells with α-Syn for 24 h. Therefore, the constant influx of α-Syn into the cells likely masked the extracellular release of α-Syn, as the observed decrease in α-Syn in pericytes could be attributed to both degradation and extracellular release. Thus, to investigate the role of extracellular release, a potential additional clearance pathway, we separately evaluated intracellular degradation and extracellular release of α-Syn. Intracellular levels of α-Syn in pericytes decreased in a time-dependent manner after α-Syn uptake. However, despite extending the exposure time for imaging, α-Syn was not detected in pericyte-derived supernatants. Therefore, we concluded that extracellular release of α-Syn could be ruled out as a mechanism for the observed decrease in α-Syn in pericytes. These findings suggest that pericytes internalize and clear α-Syn primarily through protein degradation systems rather than by extracellular secretion. Consistently, our results indicated that RBECs and astrocytes exerted insufficient α-Syn clearance ability.

To investigate the observed reduction in α-Syn in pericytes, we investigated the mechanism by which pericytes degrade α-Syn up to 6 h after α-Syn uptake. When the ALP in pericytes was inhibited by bafilomycin A1, oligomeric and monomeric α-Syn levels increased significantly. Unlike in the 24 h incubation of α-Syn with MG132, inhibition of the UPS in pericytes by MG132 did not lead to an increase in α-Syn levels. These results suggest that the ALP is predominantly involved in the degradation of both oligomeric and monomeric α-Syn during the early phase following α-Syn uptake by pericytes. This is likely due to differences in substrate specificity between the UPS and ALP. The UPS is a highly selective pathway that requires the identification and ubiquitination of target proteins via specific recognition sites, followed by the recognition and degradation of ubiquitinated proteins by the proteasome. It primarily degrades relatively low molecular weight proteins, such as IκB and p53, and plays a crucial role in maintaining cellular homeostasis, stress response, and cell cycle regulation [51]. In contrast, the ALP does not require substrate recognition sites and non-specifically degrades organelles and high molecular weight proteins [52,53]. Thus, our findings suggest that the UPS may not be as efficient in degrading high molecular weight aggregated proteins such as α-Syn in the early stages, as ubiquitination and proteasomal degradation require more time. Although both the UPS and ALP can recognize α-Syn as a substrate, the latter pathway likely acts more rapidly on the protein. Previous studies have shown that α-Syn clearance in human pericytes is mediated only by the ALP [39]. Our study is the first to demonstrate that both the ALP and UPS are involved in α-Syn clearance in healthy pericytes, despite differences in species of cells (human vs. rat) compared to previous research [39].

Based on the presented results, both monomeric and oligomeric α-Syn were cleared in pericytes via similar mechanisms. Therefore, in the following experiments, we evaluated monomeric α-Syn using recombinant human α-Syn. Next, we focused on identifying the molecules involved in α-Syn degradation by pericytes, specifically evaluating enzyme (cathepsin D) and familial PD-related molecules (DJ-1 and LRRK2). Cathepsin D (CTSD) is the principal lysosomal aspartate protease involved in α-Syn degradation in neurons [47,54]. DJ-1 is a multifunctional protein encoded by the gene *PARK7* (peroxiredoxin-like peroxidase), with mutations identified in familial PD [55]. DJ-1 primarily suppresses α-Syn aggregation through the activation of chaperone-mediated autophagy and provides a neuroprotective effect via its antioxidant activity [56]. *LRRK2* (Leucine-rich repeat kinase 2) is a commonly mutated gene that emerges in both familial and sporadic PD [57]. There is limited experimental evidence demonstrating a direct relationship between *LRRK2* and α-Syn; however, it has been suggested that *LRRK2* may indirectly influence α-Syn through protein degradation pathways, such as the ALP [58,59,60]. We found that Ctsd, DJ-1, and Lrrk2 are expressed in BBB-constituting cell types. Pericytes expressed these molecules; however, knockdown of these molecules did not confer any significant changes in α-Syn clearance in pericytes. Therefore, these results suggested that these molecules were not involved in α-Syn clearance by pericytes. Further investigations are needed to clarify the role of these molecules at the BBB in the pathogenesis of PD.

The ALP and UPS are regulated by transcriptional factors, which serve as downstream targets for several intracellular signaling pathways [61,62,63]. These regulatory mechanisms are crucial for maintaining intracellular protein quality control, ensuring complete degradation of misfolded or damaged proteins. Further, as an additional approach to identify molecular targets involved in α-Syn degradation in pericytes, we investigated signaling pathways that initiate α-Syn degradation in pericytes. We demonstrated that α-Syn activated the Akt, ERK, and p38 MAPK signaling pathways in pericytes. To determine whether these signaling pathways are involved in α-Syn degradation in pericytes, we examined the effects of specific inhibitors targeting each signaling pathway. We found that LY294002, U0126, and SB203580 significantly inhibited degradation of intracellular α-Syn. Therefore, our findings suggest that α-Syn degradation in pericytes is mediated through the Akt, ERK, and p38 MAPK signaling pathways. Studies of microglia found that α-Syn activated the Akt and p38 MAPK signaling pathways to affect autophagy [31,64]. However, these signaling pathways were unlikely to mediate α-Syn degradation in microglia. Other studies reported that the activation of these signaling pathways induces autophagy under pathological conditions, such as starvation and oxidative stress [65,66,67,68,69,70]. Thus, although the precise signaling pathways involved in autophagy-mediated α-Syn degradation in pericytes require further detailed investigation, our results suggest that pharmacological activation of the Akt, ERK, and p38 MAPK pathways could enhance α-Syn degradation by pericytes. This presents a potential novel therapeutic strategy targeting α-Syn clearance.

The BBB disruption observed in PD patients can participate in the progression of pathogenesis. Several in vitro studies have demonstrated the ability of α-Syn to disrupt the BBB. Preformed fibril α-Syn contributes to the impairment of barrier integrity of the BBB through directly activating brain microvascular endothelial cells (BMECs) to downregulate tight junction-associated proteins [71,72]. BMECs took up preformed fibril α-Syn by endocytosis, which resulted in cell death and reduced expression of tight junction-associated proteins [73]. We previously showed that monomeric α-Syn was taken up by BMECs without any changes in permeability. However, monomeric α-Syn activated pericytes to induce impaired barrier integrity of BMECs [41]. In addition, intravenous monomeric and aggregated α-Syn can cross the healthy BBB without disruption [74], although the exact α-Syn transport mechanism remains unknown. An immunohistochemical study of α-Syn in healthy human brains showed α-Syn immunoreactivity in brain endothelial cells but not in capillary walls, such as pericytes [75], whereas perivascular α-Syn immunoreactivity was detected in PD brains [71]. These findings suggest that pericytes in the healthy brain cleared α-Syn. Considering these findings, our results suggest that pericytes may clear α-Syn that penetrates BMECs to prevent α-Syn deposition in the brain parenchyma.

The α-Syn clearance ability of pericytes derived from PD patients is likely to be impaired, as suggested by a previous study [39]. Therefore, our findings that the activation of the Akt, ERK, and p38 MAPK signaling pathways mediates α-Syn degradation in healthy pericytes could partly clarify the mechanisms underlying impaired α-Syn clearance. These insights may also provide therapeutic targets for restoring α-Syn clearance ability in pericytes in patients with PD. In this context, exploring therapeutic drugs that can cross the BBB for enhancing the α-Syn clearance ability in pericytes would be a potential therapeutic strategy. Moreover, supplementation of pericytes with α-Syn clearance capability to the brain may be an effective approach.

This study has some limitations. First, we investigated the mechanism of monomeric and oligomeric α-Syn clearance in pericytes using rat-derived healthy cells. To better understand the role of pericytes in the pathogenesis of PD, future studies should address species differences in α-Syn clearance mechanisms using human-derived pericytes. Additionally, evaluating α-Syn clearance by pericytes under disease conditions—using animal models of PD or pericytes derived from PD patients or animals—will provide more insights. Furthermore, it is essential to assess the clearance capacity of pericytes for higher molecular weight and more toxic species of α-Syn, such as fibrils and phosphorylated α-Syn. Although we did not identify the specific enzymes or proteasomal components that mediate α-Syn degradation in pericytes, identifying the molecules involved in α-Syn degradation in pericytes is important for the identification of therapeutic targets and diagnostic markers, which enable early diagnosis and treatment. Furthermore, we did not investigate the receptors or binding sites for α-Syn internalization into pericytes and the subsequent activation of intracellular signaling pathways. In microglia, extracellular α-Syn interacted with Toll-like receptor (TLR) 2 or 4, triggered the activation of NF-kB, and then induced α-Syn degradation by autophagy [31]. Studies using microglia and astrocytes isolated from TLR4-knockout mice showed that TLR4 is involved in α-Syn-induced activation of microglia and astrocytes but not in α-Syn internalization [31,76]. Melatonin receptor 1 regulated LC3-associated phagocytosis, which is involved in α-Syn clearance by microglia [77]. Further research is required to identify the cell surface receptors or binding sites in pericytes that interact with α-Syn.

## 4. Materials and Methods

### 4.1. Cell Culture

All experiments were approved by the Laboratory Animal Care and Use Committee of Fukuoka University (Approval number: 2204002 and 2315117). All animal experiments complied with the ARRIVE guidelines and were conducted in accordance with the National Institutes of Health Guidelines for the Care and Use of Laboratory Animals (NIH Publications No. 8023, revised 1978).

Primary cultures of pericytes and RBECs were prepared as previously described [41,78]. Cells were isolated from 3–4-week-old Wistar rats. Astrocytes were prepared from 1–2 day old Wistar rats, as previously described [79]. Pericyte medium (DMEM (Wako, Osaka, Japan; 048-29763) supplemented with 20% FBS (Gibco/Thermo Fisher Scientific, Waltham, MA, USA; A3160602), penicillin (100 units/mL), streptomycin (100 µg/mL; penicillin–streptomycin mixed solution, Nacalai Tesque, Kyoto, Japan; 09367-34) and gentamicin (50 μg/mL, Biowest, Nuaillé, France; L0012)), RBEC medium (Dulbecco’s Modified Eagle Medium/F12 (DMEM/F12; Wako, 042-30555) supplemented with 10% fetal bovine serum (FBS; Biosera, Kansas, MO, USA; FB-1365/500), basic fibroblast growth factor (1.5 ng/mL, R&D, Minneapolis, MN, USA; 2099-FB-025), heparin (100 μg/mL, Sigma, St. Louis, MO, USA; H3149), insulin (5 μg/mL), transferrin (5 μg/mL), sodium selenite (5 ng/mL; insulin–transferrin–sodium selenite media supplement, Sigma; I1884), penicillin (100 units/mL), streptomycin (100 µg/mL; penicillin–streptomycin mixed solution, Nacalai Tesque), gentamicin (50 μg/mL, Biowest, L0012-100), hydrocortisone (500 nM, Sigma; H0135)), and astrocyte medium (DMEM (Wako) supplemented with 10% FBS (Biowest, S1820-500), penicillin (100 units/mL), streptomycin (100 µg/mL; penicillin–streptomycin mixed solution, Nacalai Tesque), and gentamicin (50 μg/mL, Biowest)) were used for maintaining pericytes, RBECs, and astrocytes, respectively.

### 4.2. α-Synuclein

Recombinant human α-Syn was prepared as previously described [80]. HiLyte488-conjugated human recombinant α-Syn (HL-α-Syn) was purchased from AnaSpec Inc. (Fremont, CA, USA; AS-55457).

### 4.3. α-Syn Uptake by Pericytes, RBECs, and Astrocytes

Primary cultures of pericytes, RBECs, and astrocytes were seeded at 10–20 × 10^4^ cells per 35 mm dish. After the cells reached confluency, pericytes, RBECs, and astrocytes were treated with or without recombinant human α-Syn or HL-α-Syn (1 μg/mL each) for 1 or 24 h in serum-free medium or physiological buffer (141 mM NaCl, 4 mM KCl, 2.8 mM CaCl_2_ anhydrous, 1 mM MgSO_4_ 7H_2_O, 1 mM NaH_2_PO_4_ 2H_2_O, 10 mM D(+)-glucose, 10 mM HEPES, pH 7.4). To remove α-Syn that binds to the cell surface, cells were washed once with 1 mL of acid wash buffer (28 mM sodium acetate, 120 mM NaCl, 20 mM sodium barbital, pH 3.0) at 4 °C. Then, cells and the collected supernatants (1000 µL) were subjected to α-Syn measurement using Western blotting.

### 4.4. Drug Susceptibility Testing

Pericytes were treated with bafilomycin A1 (10–100 nM, Selleck, Houston, TX, USA; S1039), rapamycin (10–100 nM, Selleck; S1413), MG132 (10–100 nM, EMD Millipore Corporation, Billerica, MA, USA; 474790), LY294002 (10 μM, Tocris, Ellisville, MO, USA; 1130/5), U0126 (10 μM, Tocris; 1144/5), and SB203580 (10 μM, Tocris; 1202/1) in serum-free DMEM or physiological buffer for the indicated time at 37 °C. Drugs were dissolved in dimethyl sulfoxide (DMSO, Wako; 045-24511) to prepare stock solutions. Stock solutions were diluted with serum-free DMEM or physiological buffer to a final concentration of 0.1% DMSO. An equal volume of DMSO was diluted with appropriate media for the vehicle-treated control.

### 4.5. siRNA Transfection

siRNA for rat cathepsin D (50 nM, siCtsd, Silencer^®^ Select Pre-designed siRNA, Ambion^®^/Invitrogen, Carlsbad, CA, USA; 4390771), rat DJ-1 (10 nM, siDJ-1, TriFECTa^®^ DsiRNA Kit, Integrated DNA Technologies, Coralville, IA, USA), and rat Lrrk2 (25 nM, siLrrk2, Dharmacon^TM^ siGENOME SMARTpool, Horizon Discovery Ltd., Cambridge, UK) was used. Silencer^®^ Select Negative Control (siNC, 50 nM, Life Technologies/Thermo Fisher Scientific; 4390843), Negative Control DsiRNA (DsiNC, 10 nM, Integrated DNA Technologies; 51-01-14-03), or Dharmacon^TM^ siGENOME Non-Targeting Pool #2 (siNT, 25 nM, Horizon Discovery Ltd.; D-001206-14-05) was used as an siRNA negative control. Pericytes were transfected with various siRNAs using Lipofectamine^TM^ RNAiMAX Transfection Regent (Invitrogen by Thermo Fisher Scientific; 13778075), according to the manufacturer’s protocol. siRNA–lipid complex containing siRNA and Lipofectamine^TM^ RNAiMAX (4 μL) was prepared in Opti-MEM^TM^ I Reduced Serum Medium (Gibco/Thermo Fisher Scientific; 31985062). Pericytes were incubated with siRNA–lipid complex in pericyte medium for 72 h. After transfection, pericytes were subjected to uptake experiments, Western blotting, or RT-qPCR.

### 4.6. Sample Preparation for Western Blot Analysis

Cells were lysed with 100 μL of lysis buffer (10 mM Tris-HCl, pH 6.8, 100 mM NaCl, 1 mM EDTA, pH 8.0, 1 mM EGTA, 10% glycerol, 1% Triton X-100, 0.1% SDS, 0.5% sodium deoxycholate, 2 mM Na_3_VO_4_, 1 mM sodium fluoride, 20 mM sodium pyrophosphate decahydrate, and 50 μg/mL phenylmethanesulfonyl fluoride) containing 1% phosphatase inhibitor cocktail 2 (Sigma-Aldrich), 1% phosphatase inhibitor cocktail 3 (Sigma-Aldrich), and 1% protease inhibitor cocktail (Sigma-Aldrich)). Cell lysates were stored at −80 °C until use. The total protein concentration in the cell lysates was determined using a BCA Protein assay kit (Thermo Fishier Scientific, Rockford, IL, USA), according to the manufacturer’s protocol. Aliquots (350 μL) of the supernatants were concentrated to approximately 25 μL using Amicon^®^ Ultra-0.5 Centrifugal Filter Devices (3 kDa cut-off, Millipore Corporation, Billerica, MA, USA; UFC500396). The filter units were pre-coated with 1% bovine serum albumin in PBS to minimize α-Syn adsorption.

### 4.7. Western Blot Analysis

Samples were mixed with 3× sample buffer (0.03% bromophenol blue, 6% sodium dodecyl sulfate (SDS), 30% glycerol, 187.5 mM Tris-HCl) or 6× sample buffer (Nacalai Tesque; 09500-64) containing 2-mercaptethanol and boiled at 95 °C for 5 min. Equal amounts of protein were electrophoretically separated on 13 or 15% SDS-polyacrylamide gels (0.03 A/gel, 50–60 min). After electrophoresis, the proteins were transferred to PVDF membranes (ClearTrans^®^ SP; Wako; 033-22453) in transfer buffer (24.8 mM Tris HCl, 191.8 mM glycine, 20% methanol; 100 V, 180 min). Membranes for α-Syn detection were incubated with 4% paraformaldehyde (Wako) for 15 min at room temperature. Then, the membranes were blocked with Blocking One (Nacalai Tesque; 03953-95) or Blocking One-P (Nacalai Tesque; 05999-84) for phosphorylated proteins for 30 min at room temperature. The membranes were incubated with primary antibodies diluted in 5% Blocking One or Blocking One-P in Tris-buffered saline (10 mM Tris HCl, pH 8.0, 136 mM NaCl, 2 mM sodium orthovanadate, 50 mM sodium fluoride, pH 7.85) containing 0.1% Tween 20 (TBS-T) overnight at 4 °C. α-Syn, p62, LC3, cathepsin D, DJ-1, Lrrk2, and β-actin were detected using anti-α-synuclein antibody (MJFR1) (ab138501, 1:1000; Abcam plc, Cambridge, UK), anti-SQSTM1/p62 antibody (ab56416, 1:1000; Abcam plc), LC3B Rabbit antibody (2775S, 1:1000; Cell Signaling Technology, Danvers, MA, USA), cathepsin D (E179) Rabbit antibody (69854S, 1:1000; Cell Signaling Technology), DJ-1 (D29E5) XP^®^ Rabbit mAb (5933S, 1:1000; Cell Signaling Technology), anti-LRRK2 antibody (MJFF2) (ab133474, 1:1000; Abcam plc), and anti-β-actin antibody (clone CA-15; A1978, 1:8000; Sigma-Aldrich, MO, USA). Phosphorylation of p42/p44 MAPK (Erk), p38 MAPK, and Akt was detected using primary antibodies against phospho-p44/42 MAPK (Erk1/2) (Tyr202/Tyr204) (D13. 14. 4E) XP^®^ Rabbit mAb (4370S, 1:1000; Cell Signaling Technology), phospho-p38 MAPK (Tyr180/Tyr182) (D3F9) XP^®^ Rabbit mAb (4511S, 1:1000; Cell Signaling Technology), and phospho-Akt (Tyr308) (C31E5E) Rabbit mAb (2965S, 1:1000; Cell Signaling Technology). The membrane was washed three times with TBS-T for 10 min and then incubated with horseradish peroxidase-conjugated anti-mouse IgG or anti-rabbit IgG (170-6516 or 170-6515, 1:5000 or 10,000; BIO-RAD, Hercules, CA, USA) diluted in 5% Blocking One in TBS-T for 1 h at room temperature. The membrane was washed five times with TBS-T for 5 min, and target protein bands were visualized using a Clarity Western ECL Substrate (BIO-RAD). To re-probe total p42/p44 MAPK, p38 MAPK, and Akt, membranes were incubated in stripping buffer (0.2 M glycine, 0.1% SDS and 1% Tween 20, pH 2.2) for 15 min, and this step was repeated. Total p42/p44 MAPK, p38 MAPK, and Akt were detected using primary antibodies against p44/42 MAPK (Erk1/2) (137F5) Rabbit mAb (4695S, 1:1000; Cell Signaling Technology), p38 MAPK antibody (9212S, 1:1000; Cell Signaling Technology), and Akt (pan) (C67E7) Rabbit mAb (4691S, 1:1000; Cell Signaling Technology). The band images were digitally captured using a Multi Imager II ChemiBOX (BioTools, Gunma, Japan), and band intensities were quantified using the ImageJ 1.48 software (NIH, Bethesda, MD, USA).

### 4.8. Real-Time Quantitative PCR

Total RNA was extracted from cells using the FastGene RNA Basic Kit (FastGene Co., Ltd., Tokyo, Japan; FG-80250), according to the manufacturer’s protocol. Equivalent amounts of RNA from each sample were reverse-transcribed with FastGene cDNA Synthesis 5× ReadyMix OdT (FastGene Co., Ltd.; NE-LS65). Real-time PCR was conducted in a Light Cycler 96 System (F. Hoffmann-La Roche, Ltd., Basel, Switzerland) using a KAPA SYBR Fast qPCR Kit (Kapa Biosystems, Inc., London, UK; KK4602), according to the manufacturer’s protocol. After pre-incubation at 95 °C for 3 min, PCR was performed using the following conditions: 45 cycles of 95 °C for 10 s, 60 °C for 20 s, and 72 °C for 1 min, using specific primers (purchased from Takara Bio Inc. (Shiga, Japan), Sigma, and Integrated DNA Technologies, Inc.) to amplify the genes of interest. Primer sequences were as follows: Cathepsin D (Ctsd; NM_134334, Integrated DNA Technologies, Forward: 5′-TGACAAGTCCAGCACCTATG-3′, Reverse: 5′-TTCTCCACCTTGATACCTCCTA-3′), DJ-1 (Park7; NM_057143, Sigma, Forward: 5′-AAGGACAAAATGATGAACGG-3′, Reverse: 5′-CTCTCTAGTCTTTGAGAACAAG-3′), Lrrk2 (NM_001191789, Integrated DNA Technologies, Forward: 5′-GACCTTCATTCCCGACTCTTC-3′, Reverse: 5′-GTCCCTGTAAACTTCTCCTACAC-3′). Ywhaz (NM_013011, Takara, Forward: 5′-GAGTCGTACAAAGACAGCACGCTAA-3′, Reverse: 5′-GTGGGACAGCATGGATGACAA-3′). Ywhaz was used as the reference gene; therefore, the threshold cycle value (Cq) of the target genes was normalized to that of Ywhaz. Total RNA isolated from rat brain was purchased from Clontech Laboratories, Inc. (Mountain View, CA, USA; Premium Total RNA, Rat Brain, cerebral cortex; 636677) and used as a calibrator for relative quantification of gene expression.

### 4.9. Statistical Analysis

The results are expressed as the means ± standard error of the mean (SEM). All data were assessed and statistically analyzed using GraphPad Prism 10.1.2 (GraphPad Software, San Diego, CA, USA). Statistical differences between groups were analyzed using one-way analysis of variance (ANOVA) with Dunnett’s multiple comparisons test or the Tukey–Kramer test. The unpaired *t*-test was applied to compare two groups. *p* < 0.05 was considered statistically significant.

## 5. Conclusions

We demonstrated that pericytes possess the ability to degrade α-Syn via Akt, ERK, and p38 MAPK signaling pathway activation. In pericytes, α-Syn degradation is mediated by the ALP and UPS. Notably, this α-Syn clearance ability in pericytes was more efficient than that observed in other BBB-constituting cells, such as brain endothelial cells and astrocytes, suggesting a pericyte-specific role in α-Syn clearance at the BBB. Our findings highlight the importance of α-Syn clearance by pericytes in understanding the pathogenesis and progression of PD. This mechanism serves as a potential therapeutic target for disease-modifying therapies aimed at preventing the propagation of α-Syn pathology in the brain.

## Data Availability

The original contributions presented in this study are included in the article. Further inquiries can be directed to the corresponding author.

## Abbreviations

The following abbreviations are used in this manuscript:

ALP	Autophagy–Lysosome Pathway
α-Syn	α-Synuclein
BBB	Blood–Brain Barrier
Ctsd	Cathepsin D
ERK	Extracellular signal-regulated kinase
HL-α-Syn	HiLyte488-conjugated human recombinant α-Syn
MAPK	Mitogen-Activated Protein Kinase
Lrrk2	Leucine-Rich Repeat Kinase 2
MSA	Multiple System Atrophy
NC	Negative Control
PD	Parkinson’s disease
PSP	Progressive Supranuclear Palsy
RBECs	Rat Brain Endothelial Cells
TLR	Toll-Like Receptor
UPS	Ubiquitin–Proteasome System
